# Distinguishing between primary and secondary volcaniclastic deposits

**DOI:** 10.1038/s41598-019-48933-4

**Published:** 2019-08-27

**Authors:** C. Sohn, Y. K. Sohn

**Affiliations:** Department of Geology and Research Institute of Natural Science, Gyeongsang National University, Jinju, 52828 Republic of Korea

**Keywords:** Stratigraphy, Volcanology, Sedimentology

## Abstract

The distinction between primary and secondary volcaniclastic deposits, which are currently defined as the “direct” products of volcanic eruptions and the “reworked” products of the former, respectively, is the first step to interpreting volcaniclastic deposits, particularly the genetic connection with active volcanism. The distinction appears straightforward, but is not always applicable to natural deposits. During the 3.7 ka BP eruption of the Songaksan tuff ring, Jeju Island, Korea, there was an invasion of typhoon. The tuff ring was partly submerged underwater and affected by wave activity for over a day, resulting in a peculiar volcaniclastic deposit composed of both vent-derived (primary) and substrate-derived (reworked or secondary) volcaniclastic particles. We propose a new term “reprocessed” for a category of volcaniclastic deposits or particles, which originated directly from volcanic eruption but was deposited finally by nonvolcanic processes. Here we show that both reprocessed and reworked particles can coexist in the same volcaniclastic deposit, making it impossible to differentiate it into either a primary or a secondary deposit according to the current definition of volcaniclastic deposits. We thus define the secondary volcaniclastic deposits as comprising either or both of reprocessed and reworked volcaniclastic particles.

## Introduction

Volcaniclastic deposits form by various processes of volcanic and sedimentary nature, comprising a range of particles that underwent diverse fragmentation, transport, and depositional processes^1–4^. For primary volcaniclastic deposits, there have been two approaches to define them. One is to name a deposit according to the mode of fragmentation of the constituent particles^5–7^. The other is to name it based on the process of deposition^2,3,8^. The latter approach has been more widely used in academia, and has a more rational basis in that the only unique event shared by all particles within a deposit is the process of deposition whereas the individual particles in the deposit might have originated from different sources and experienced different fragmentation events^8^.

Volcaniclastic particles that were once deposited by volcanic processes and then “reworked” by normal sedimentary processes during and after an eruption but before the lithification of the deposit constitute secondary volcaniclastic deposits. Particles that were derived from the weathering of lithified volcaniclastic deposits and solidified lavas are neither primary nor secondary volcaniclastics but are sedimentary particles of volcanic heritage, for which the term “epiclastic” is used^2,3,5,6^. Reworking of unlithified volcaniclastic deposits is therefore a key process that forms and defines the secondary volcaniclastic deposits. Reworking is also a key element in the definition of primary volcaniclastic deposits, for which two subtly different definitions are possible. One is a restrictive definition that defines them as the direct product of an eruption, and the other is an inclusive definition that defines all un-reworked volcaniclastic deposits as the primary volcaniclastic deposits. White and Houghton^8^ supports the latter definition, stating that “all deposits that do not involve interim storage of particles are primary deposits, regardless of whether transport occurs through air, water, granular debris, or some combination thereof.” According to this definition, all volcaniclastic deposits that were finally deposited by nonvolcanic processes, such as stream flows, waves, ocean currents, winds, and etc, are primary unless the constituent particles experienced temporary deposition and reworking before final deposition. One can question, however, whether such current- or wave-worked deposits can be covered by the restrictive definition of primary volcaniclastic deposits because the final deposition of the deposits was accomplished by a process that is completely unrelated with a volcanic eruption. In order to solve this problem, we proposes some modification of the current definition of volcaniclastic deposits based on a study of a deposit in Jeju Island, Korea, that can be described as both primary and secondary deposits according to the current volcaniclastic terminology. We suggest that some deposits or particles, which originated directly from volcanic eruption but was deposited finally by nonvolcanic processes, can be described with a new term “reprocessed”, and that both reprocessed and reworked particles constitute secondary volcaniclastic deposits.

## Volcaniclastic Deposit at Songaksan

**Figure 1:**
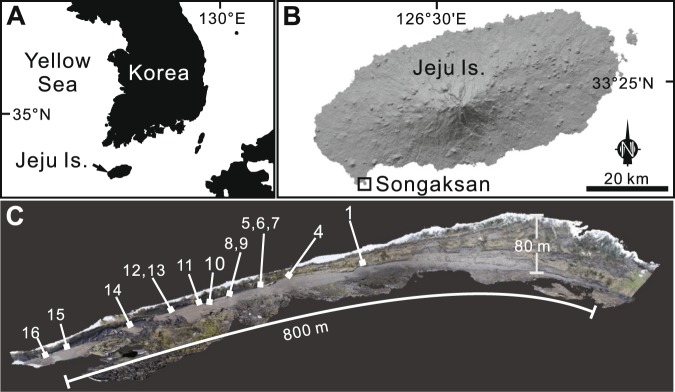
Location of the study area. (**A**) Location of Jeju Island. (**B**) Digital elevation model of Jeju Island volcano, having typical shield morphology. (**C**) 3D model of the exposures along the western coast of the Songaksan tuff ring with the locations of sedimentological observations, which was built by combining hundreds of still images taken with a hand-held camera, using a commercial software Agisoft PhotoScan Professional version 1.4.3.

**Figure 2:**
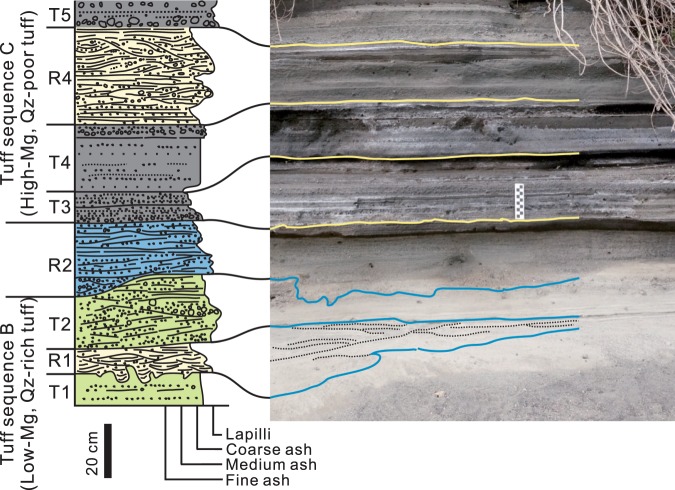
Deposit stratigraphy. Songaksan tuff ring consists of four tuff sequence (A to D). Storm wave-worked units (R1, R2 and R4) occur along and adjacent to the boundary between tuff sequences B and C), which have contrasting tephra compositions and accidental componentry.

Jeju Island is an intraplate alkali basaltic volcano built on the southeastern Yellow Sea continental shelf^9,10^ (Fig. 1A). Songaksan is a young phreatomagmatic volcano, which erupted ~3.7 ka BP at the southwestern coast of the island^11,12^ (Fig. 1B), providing a complete cross-section of a tuff ring (Fig. 1C). Recent studies reveal that the tuff ring resulted from a single continuous eruption^13^ possibly in a month when the sea level was almost identical to that at present^14^. The tuff ring formed mostly above high tide level by pyroclastic surges and fall^15^, but contains three interbeds of horizontally laminated, low- to high-angle cross-stratified, and hummocky to swaly cross-stratified volcaniclastic deposits in the middle of the tuff sequence (Fig. 2) up to an altitude of ~5.5 m, i.e., ~4.5 m above the high tide level. These interbeds, named units R1, R2, and R4, are interpreted to have formed by wave activity in a swash to surf zone when the sea level rose several meters above normal high-tide level during a storm event^16^. The triple intercalation of the wave-worked deposits is interpreted to reflect three tidal cycles during the storm event that is inferred to have lasted ~1.5 day. In this paper, we focus on unit R2 because it occurs along an interface between two tuff sequences that have contrasting accidental componentry and juvenile tephra composition, thereby making it possible to assess the relative proportions of the vent-derived (i.e., primary) and substrate-derived (i.e., reworked or secondary) particles in the deposit.

**Figure 3:**
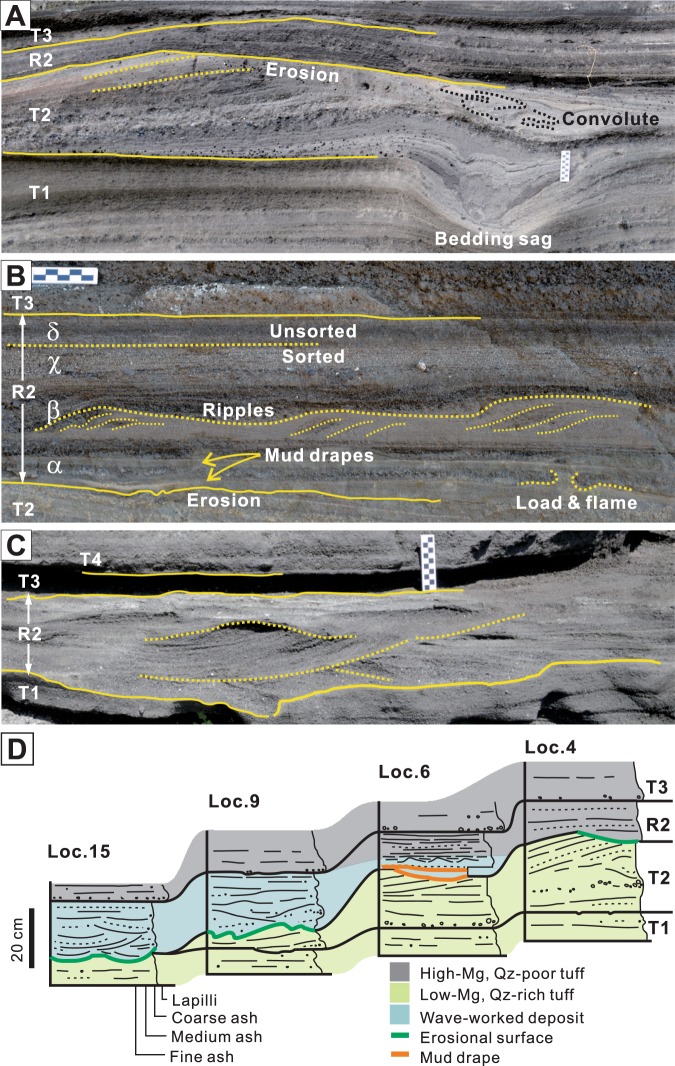
Deposit features of unit R2. (**A**) Primary facies of unit R2 at loc. 4, consisting of poorly sorted and crudely stratified tuff. The underlying unit T2 shows well-developed megaripple bedforms and bedding sags, and is truncated and deformed by storm waves. (**B**) Transitional facies of unit R2 at loc. 6, consisting of well sorted sand (ash) with mud drapes (α), seaward-migrating ripple formset with internal high-angle cross-stratification (β), horizontally laminated well-sorted sand (χ), and poorly sorted and crudely stratified tuff (δ), suggesting gradual emergence of the depositional site from a surf zone. (**C**) Swaly cross-stratified facies of unit R2 at loc. 15. (**D**) Columnar sections of unit R2 and adjacent units at selected localities, showing lateral transition of primary and wave-worked deposits within the same depositional unit.

Detailed sedimentological observations of unit R2 reveal that the unit comprises both primary and wave-worked deposits. The former consists of poorly sorted and crudely stratified (lapilli) tuff and occurs in the proximal part (to the east of loc. 4; Fig. 1C), at altitudes higher than ~5.5 m above sea level, and at a distal locality (loc. 14), where the deposit accumulated upon a lava bulge, about 1 m higher than the surrounding areas. Stratification in these deposits is generally planar but shows subtle undulations and thickening/thinning of layers over the bedform reliefs and other topographic irregularities of the underlying unit T2 (Fig. 3A). The overall deposit features and its lateral continuation into thicker and coarser-grained deposit toward the crater rim suggest primary deposition from a pyroclastic surge^15^.

At altitudes between ~5.0 and 5.5 m (between loc. 4 and 7), unit R2 shows peculiar vertical facies changes from (1) well sorted very fine sand (ash) intercalated with mud drapes at the base, (2) seaward-migrating ripple cross-laminated and high-angle cross-stratified deposit composed of well-sorted sandy to granular materials, (3) horizontally laminated deposit composed of well-sorted sandy materials, to (4) poorly sorted and crudely stratified deposit at the top (Fig. 3B). The facies units 1 and 2 are interpreted to have formed in the surf zone affected by storm waves and return flows driven by coastal setup together with periodic suspension settling of fines^16^. The facies unit 3 suggests deposition on a beach face by swash and backwash of breaking waves; the facies unit 4 is interpreted to be primary pyroclastic surge deposit. The facies transition at these localities suggests gradual emergence of the depositional site from a surf zone to a subaerial surface associated with falling sea level during the ebb tide^16^.

At other distal localities in lower altitudes, unit R2 comprises hummocky to swaly cross-stratified deposits passing upward into horizontally to low-angle stratified deposits (Fig. 3C), suggesting deposition by wave-induced combined flows in the surf zone followed by deposition on a beach face by breaking waves^16^.

The coexistence of evidently primary volcaniclastic deposits and wave-worked deposits and their lateral transition within the same depositional unit (Fig. 3D) provide strong evidence for the contemporaneity of volcanic eruption and storm wave-working of volcaniclasts during deposition of unit R2. In addition, the laterally extensive erosion of the underlying unit T2 suggests partial incorporation of unit T2 tephra into unit R2. We thus attempt to assess the relative proportions of the tephra that was reworked from unit T2 (i.e., secondary volcaniclasts) and the tephra that was derived from contemporaneous eruption (i.e., primary volcaniclasts) based on the analyses of the chemistry of juvenile tephra particles and the accidental componentry.

**Figure 4:**
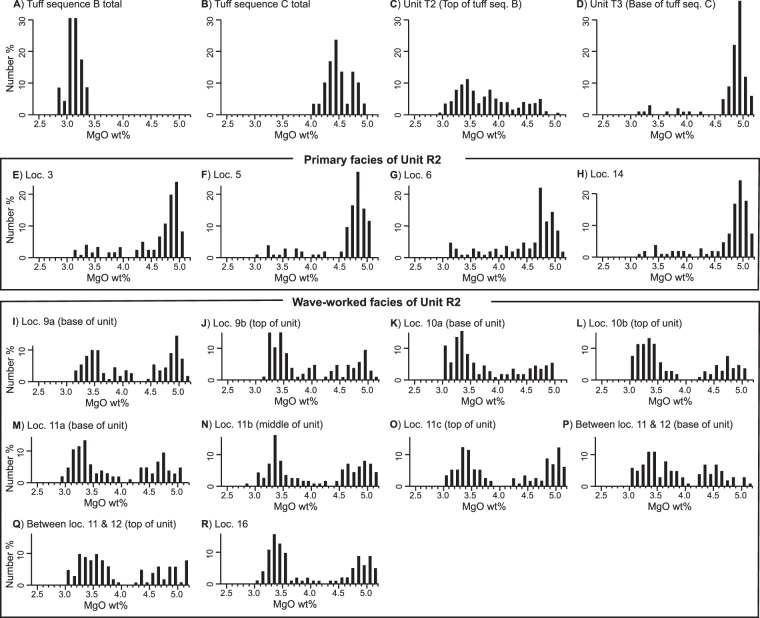
Distribution of MgO contents. (**A**) Tuff sequence B (excluding unit T2). (**B**) Tuff sequence C. (**C**) Unit T2. (**D**) Unit T3. (**E**–**H**) Primary facies of unit R2. (**I**–**R**) Wave-worked facies of unit R2. The MgO contents of tuff sequences B and C (**A**,**B**) were recalculated from the original data of Go *et al*.^13^, in which the magnesium contents were given in magnesium number.

Previous studies reveal that the Songaksan tuff ring can be subdivided into four tuff sequences (A to D), which resulted from four magma pulses with marked chemical variations, particularly across the boundary between the tuff sequences B and C^13,17^. Above all, MgO content is useful for distinguishing juvenile particles from different tuff sequences, as is the case in other mafic volcanoes^18,19^. The MgO contents of juvenile tephra particles from tuff sequence B range between 2.83 and 3.33 wt% with a mean at 3.13 wt% (Fig. 4A), whereas those from tuff sequence C range between 4.00 and 4.90 wt% with a mean at 4.42 wt%^13^ (Fig. 4B).

In order to obtain more representative values of MgO contents of unit T2 tephra, we analyzed the chemical composition of 342 juvenile particles from the unit (Table 1). The analysis shows that the tephra particles of unit T2 have a wide range of MgO contents with some tephra particles having intermediate MgO contents (Fig. 4C). We interpret that the tuff unit comprises (1) low-Mg tephra (MgO content ≤ 3.33 wt%) from the earlier magma batch that was left in the diatreme, (2) high-Mg tephra (MgO content ≥ 4.00 wt%) from the later magma batch, and (3) intermediate-Mg tephra (3.33 wt% <MgO content <4.00 wt%) which resulted from mixing of the two magmas in the feeder dike. Geochemical and petrographic evidence for the magma mixing in Songaksan volcano is provided in a former publication^20^, and similar magmatic processes are also reported in other monogenetic volcanoes^21,22^. The analysis shows that unit T2 comprises ~30% low-Mg tephra, ~27% high-Mg tephra, and ~43% intermediate-Mg tephra (Table 1). A simple mathematical calculation yields a solution that ~55% of low-Mg magma and ~45% of high-Mg magma contributed to form unit T2.

**Table 1:** MgO contents of juvenile tephra particles and the contents of accidental quartz grains in units T2 and R2 at Songaksan tuff ring.

Unit	T2	R2	R2	R2	R2	R2	R2	R2	R2	R2
Location	Loc. 1	Loc. 3	Loc. 5	Loc. 6	Loc. 9	Loc. 10	Loc. 11	Loc. 11′*	Loc. 14	Loc. 16
Facies	Primary	Primary	Primary	Primary	Wave-worked	Wave-worked	Wave-worked	Wave-worked	Primary	Wave-worked
Number of juvenile particles	342	123	104	105	219	219	333	203	108	103
Average MgO (wt%)	3.70	4.57	4.63	4.50	3.97	3.78	3.96	3.82	4.61	3.90
Low-Mg tephra (%)	29.82	4.88	5.77	7.62	22.37	33.79	31.83	29.06	3.70	33.01
High-Mg tephra (%)	26.90	81.30	85.58	80.00	42.92	32.42	45.05	37.44	85.19	39.81
Intermediate-Mg tephra (%)	43.27	13.82	8.65	12.38	34.70	33.79	23.12	33.50	11.11	27.18
T2-derived tephra (%)	—	—	—	—	62	89	64	82	—	72
Vent-derived tephra (%)	—	—	—	—	38	11	36	18	—	28
Accidental quartz (%)	34.31	1.50	7.10	4.30	26.61	32.15	19.31	26.25	1.80	8.50

^*^Loc. 11**′** is located between loc. 11 and 12.

As for unit R2, similar analyses were performed for 1,517 particles from 9 localities (Table 1). The distribution of MgO contents of individual tephra particles is strongly dependent on the deposit facies. The primary deposits mostly comprise high-Mg tephra, of which the MgO contents are higher than the average MgO content of tuff sequence C but are similar to those of unit T3 (Fig. 4D–H). The tephra composition thus attests to derivation of the tephra mostly from the later high-Mg magma and subordinately from the diatreme-filling low-Mg tephra. On the other hand, wave-worked deposits have strongly bimodal distribution of tephra composition (Fig. 4I–R), attesting to their derivation from both the underlying unit T2 and the newly erupted high-Mg magma. A simple mathematical calculation suggests that ~60 to 90% of tephra in these deposits originated from the reworking of unit T2, and the rest from contemporaneous eruption of high-Mg magma (Table 1).

The contents of accidental particles, composed mostly of detrital quartz grains from subsurface sedimentary strata, provide further evidence for the dual sources of unit R2 tephra. Unit T2 contains abundant accidental particles (34.3% quartz plus minor amounts of lithics and other crystals) (Table 1), whereas the tuff sequence C contains much smaller amount (an average of 8.0%) of accidental particles^13^. The componentry analysis shows that the primary facies of unit R2 contains less than 10% quartz, whereas the wave-worked facies contains ~19 to 32% quartz (Table 1), suggesting that the wave-worked facies of unit R2 comprises significant proportions of both vent-derived and substrate-derived tephras whereas the primary facies of the unit comprises only vent-derived tephra.

## Discussion

Volcaniclastic materials that were emitted from a vent are transported through air, water, granular debris or some combination thereof^8^, as witnessed in recent eruptions of Mount St. Helens, Washington, 1980^23^, Nevado del Ruiz, Columbia, 1985^24^, and Soufrière Hills volcano, Montserrat, 2003^25^ among others. In addition to the changes in the transport medium, they can be subject to different processes and deposited finally by a process that is commonly completely unrelated with a volcanic eruption, such as streamflows, ocean currents^26^, waves/tsunamis^27^, tidal currents^28^, and winds^29,30^. The term “resedimented syn-eruptive volcaniclastic” was formerly proposed to describe a class of similar volcaniclastic deposits that are syn-eruptive but are not, or do not appear to be, primary^3^. However, those deposits resulting from such syn-eruptive, hybrid or combined volcanic-sedimentary processes have been poorly explored so far, and have been largely regarded as deposits of uncertain origin or ambiguous deposits^8^. Currently, deposits of such hybrid processes are defined as primary volcaniclastic deposits as long as the volcanic materials did not involve interim storage and reworking^8^.

The wave-worked facies of unit R2 is evidently syn-eruptive because it passes laterally into a primary facies toward the crater rim, and is interpreted to have formed by the entrance of a pyroclastic current into a stormy sea. The term “resedimented syn-eruptive” is not, however, appropriate to describe the wave-worked facies of unit R2 because only part of the deposit was resedimented from an earlier deposited primary deposit. So we propose a new term “reprocessed” for a category of volcaniclastic deposits or particles, which originated directly from a volcanic eruption but were deposited finally by nonvolcanic processes. The word “reprocess” has the meaning of “process again or differently” and seems to be an appropriate term to describe such deposits or particles. The terms such as “wave-modified but syn-eruptive” or “current-modified but syn-eruptive” can be used to describe the depositional processes of individual deposits, but the term “reprocessed” is proposed here as a comprehensive term for describing a category of syn-eruptive volcaniclastic deposits deposited finally by nonvolcanic processes, just as the term “reworked” is used, irrespective of the exact reworking processes involved.

We also propose to classify reprocessed volcaniclastic deposits as a subcategory of secondary volcaniclastic deposits because of a practical reason that such deposits would hardly be described as primary deposits by geologists because their depositional structures, which are the key criterion to distinguish between primary and secondary volcaniclastic deposits, would indicate a nonvolcanic process. The case study at Songaksan also shows clearly that reprocessed and reworked particles can coexist in the same deposit, making it practically and conceptually impossible to differentiate it into either a primary or a secondary deposit according to the current volcaniclastic terminology. We thus propose to define the secondary volcaniclastic deposits as comprising either or both of reprocessed and reworked volcaniclastic particles.

The new distinction proposed here will remove the ambiguity in the current distinction between primary and secondary volcaniclastic deposits because the final depositional processes can be more readily interpreted from a deposit than the interim processes of temporary deposition and resedimentation. According to our definition, we only need to interpret the “final” depositional processes of a deposit to distinguish between primary and secondary volcaniclastic deposits because all volcaniclastic deposits that were finally deposited by nonvolcanic processes are secondary volcaniclastic deposits. Where additional interpretation of the contemporaneity of volcanic eruption and the occurrence of interim storage and reworking of volcanic debris is possible, the secondary deposit can further be classified into a reworked or a reprocessed deposit.

## Methods

### Sedimentological observations

Sedimentological observations were made at fourteen sites along the western shore of the Songaksan tuff ring. Grain size, sorting, and clast shape, bed thickness, depositional and erosional structures, post-depositional deformation structures, bed geometry, and lateral bed continuity were described in the field. Individual beds or units were correlated by tracing them along the continuous coastal exposures. The altitudes of key stratigraphic surfaces were obtained by the South S82T RTK GPS surveying unit.

### Componentry analysis

A total of eighteen specimens were obtained from these sites. About 50 grams of each specimen were immersed in water for a day and treated in an ultrasonic vibrator for 10 min. Those fractions coarser than 4 Φ (1/16 mm) were then selected by wet sieving, dried, and dry-sieved at 1 Φ interval. The ash grains between 0 Φ (1 mm) and 1 Φ (0.5 mm) were then impregnated with epoxy and prepared for polished thin sections for componentry analysis. Backscattered electron (BSE) images were obtained from the polished sections using a JEOL JXA-8100 electron microprobe at the Center for Research Facilities of Gyeongsang National University. 125 to 182 particles were counted from the BSE images of each polished section to obtain the percentages of accidental quartz grains in each specimen.

### Chemical analysis of tephra

The MgO contents of more than 100 juvenile tephra particles from each specimen were also obtained from the same polished thin sections. A field-emission electron probe micro-analyzer (Model JXA-8530F PLUS, Jeol) at the same institute was used to obtain the MgO contents. Energy dispersive spectroscopy (EDS) analysis (Model X-max, Oxford) was conducted at 15 kV voltage, 10 nA current with the focal distance of 11 mm. The percentages of unit T2-derived and vent-derived tephras of unit R2 were calculated with the equation MgO_specimen_ = (1 − x)MgO_B_ + xMgO_C_, where MgO_specimen_ is the average MgO content of a specimen, and MgO_B_ and MgO_C_ are the average MgO contents of tuff sequences B and C, which are 3.13 wt% and 4.42 wt%, respectively. Tephra particles of the primary facies of unit R2 have MgO contents larger than MgO_C_, and all of them are interpreted to be vent-derived.

## Data Availability

The datasets generated during and/or analysed during the current study are available from the corresponding author on reasonable request.
